# Vegetation increases abundances of ground and canopy arthropods in Mediterranean vineyards

**DOI:** 10.1038/s41598-022-07529-1

**Published:** 2022-03-07

**Authors:** Chloé Blaise, Christophe Mazzia, Armin Bischoff, Alexandre Millon, Philippe Ponel, Olivier Blight

**Affiliations:** 1 Aix Marseille Univ, Avignon Université, CNRS, IRD, IMBE, Aix-en-Provence, France; 2 Avignon Université, Aix Marseille Univ, CNRS, IRD, IMBE, Avignon, France

**Keywords:** Ecology, Agroecology, Conservation biology, Restoration ecology

## Abstract

The decline of arthropod populations observed in many parts of the world is a major component of the sixth mass extinction with intensive agriculture being one of its main drivers. Biodiversity-friendly farming practices are taking centre stage in the recovery process. In vineyards, vegetation cover is commonly used for production purposes, to reduce soil compaction by machinery use and soil erosion. Here we examined the effects of vegetation cover and soil management on the abundance of ground- (spiders, beetles, Hemiptera and harvestmen) and canopy-dwelling (wild bees, green lacewings, beetles and Hemiptera) arthropods in three categories of vineyards: (i) vineyards with no vegetation, (ii) partially vegetated (every second inter-row is vegetated) and (iii) all inter-rows are vegetated. We recorded a general positive effect of a decrease in soil perturbation intensity and corresponding higher vegetation cover on arthropod abundance. Plant species richness was the most important vegetation parameter, with a positive effect on spiders, harvestmen, hemipterans and beetles (ground and canopy) abundances. Using a path analysis, we also highlighted the central role of inter-row vegetation management in trophic and non-trophic relationships between vegetation and arthropods, and between arthropod groups. Our results demonstrate the benefits of a softer soil management preserving a diverse vegetation cover for the conservation of arthropods in Mediterranean vineyards.

## Introduction

The decline of arthropod populations observed in many parts of the world over the past decades is a major component of the sixth mass extinction currently observed^1,2^. Although multi-causal, arthropod decline is strongly associated with the intensification of agricultural practices since the Green Revolution^3^. This is due to the homogenisation of landscapes leading to the loss of semi-natural habitats, the toxicity of pesticides and the mechanisation of farming practices, reducing diversity and abundance of plants on which arthropods directly depend^4,5^.

Given their role as decomposers^6,7^, herbivores^6^, predators^8^, pollinators^9,10^ and prey of many vertebrate taxa, the general decline of arthropods threatens the functioning of ecosystems in agricultural but also in natural environmental contexts. Moreover, arthropods provide ecosystem services that directly affect production (pollination, pest regulation, recycling of organic matter, bioturbation)^11,12^. Their loss is therefore expected to have serious economic consequences in addition to ecological consequences^13^.

In order to stop the loss of arthropod biomass and diversity, and their related services, various agricultural practices have been developed to increase sustainability and conservation of biodiversity and its functions within agricultural ecosystems. The restoration of semi-natural habitats in agricultural areas is a key aspect of agro-ecology^14,15^. Sowing plants to cover the soil is increasingly promoted to improve predation^16^, pest regulation^17^ and the refuge function for insect biodiversity^18,19^ in addition to soil protection against erosion^20^. The presence of plants other than the cultivated species is therefore expected to provide multiple benefits for perennial crops, such as the grapevine^21^.

Diversifying the plant community in crop production by integrating annual and perennial plant species allows arthropods to access more diverse resources and habitats^22–24^. Several studies have demonstrated the positive effect of inter-row vegetation on grapevine arthropod communities^18,25,26^. The establishment of vegetation cover is often accompanied by a general trend towards an increase in the abundance and diversity of arthropods. However, there is a high variability in the response to vegetation when looking at arthropod guilds separately. For example, Sáenz-Romo et al.^19^ reported a higher abundance of epigeic arthropods in grassy inter-rows but a lack of response of those collected in the grapevine canopy. Conversely, Eckert et al.^18^ found that epigeic arthropods were less numerous in densely vegetated inter-rows but more abundant in the adjacent canopy. These contrasting results demonstrate the need to improve the understanding of vegetation effects on arthropods, and to investigate more finely the relationships between the different components in response to the management of vegetation cover in vineyards.

In Mediterranean ecosystems known for their high level of biodiversity^27^, the integration in crop production of more sustainable agricultural practices may represent an important lever for biodiversity conservation. The objective of our study was to test the restoration of inter-row vegetation in Mediterranean vineyards as a measure for arthropod conservation. Using taxonomic and functional approaches, we studied the response of arthropods and the organisation of trophic relationships to three modes of inter-row management: mechanical soil management to remove vegetation, partially vegetated (one out of two inter-rows vegetated) and all inter-rows vegetated. We focused on two ecosystem components: the epigeic fauna (beetles, spiders, harvestmen, Hemiptera) and the flying fauna occurring in the grapevine canopy (beetles, wild bees, green lacewings and Hemiptera). We hypothesised that a dense and diversified plant cover increases the abundance of arthropods through trophic relationships between vegetation, phytophagous arthropods and predators both in the canopy and on the ground.

## Material and methods

### Study sites and design

This study was carried out in South-eastern France, between the southern slopes of the Luberon mountain and the Durance river (Appendix 1). The landscape is dominated by medium to small-sized vineyards (64% < 0.5 ha, 13% > 1 ha)^28^. This area is subject to a Mediterranean climate, with hot and dry summers followed by mild winters and rainfall mostly occurring in autumn and spring. Annual temperatures and total annual rainfall average 14.5 ± 0.1 °C and 607 mm respectively (Pertuis meteorological station, 1991–2020). In this region, most vineyards are mechanically managed between rows to keep bare soil from mid-spring to the autumn rain seasons. Permanently vegetated vineyards are mowed or laid down before summer.

In 2018, we selected 27 vineyards where volunteer farmers (*N* = 9) applied one of the three following types of inter-row management: (i) periodic mechanical soil management to remove vegetation (shallow tillage) (0/2; *N* = 8), (ii) partially vegetated (every second inter-row is vegetated for a minimum of four years) (1/2; *N* = 10) and (iii) all inter-rows are permanently vegetated (2/2; *N* = 9) (Appendix 2). The selected vineyards had an average size of 7008 ± 746 m^2^ representative of the study region and were dispersed over an area of 20 km by 6 km. According to the French guidelines for organic viticulture no chemical was applied to 23 vineyards for at least three years. In the other four vineyards, farmers use chemical fertilisers and fungicides. As none of the 27 vineyards had suffered from pest attacks, no insecticides were applied. The only insect considered as a pest in the region is *Scaphoideus titanus* (Hemiptera: Ciccadellidae) which has a limited distribution. The inter-rows of all these vineyards were sown at least once in the last five years with commercial grass-legume mixtures. However, with the exception of two vineyards, they were all dominated by non-sown spontaneously emerging plant species. All vineyards were planted with either Syrah or Grenache grapevines for more than 15 years, the most common grape varieties in this region.

### Characterisation of vegetation cover and arthropod communities

**Figure 1:**
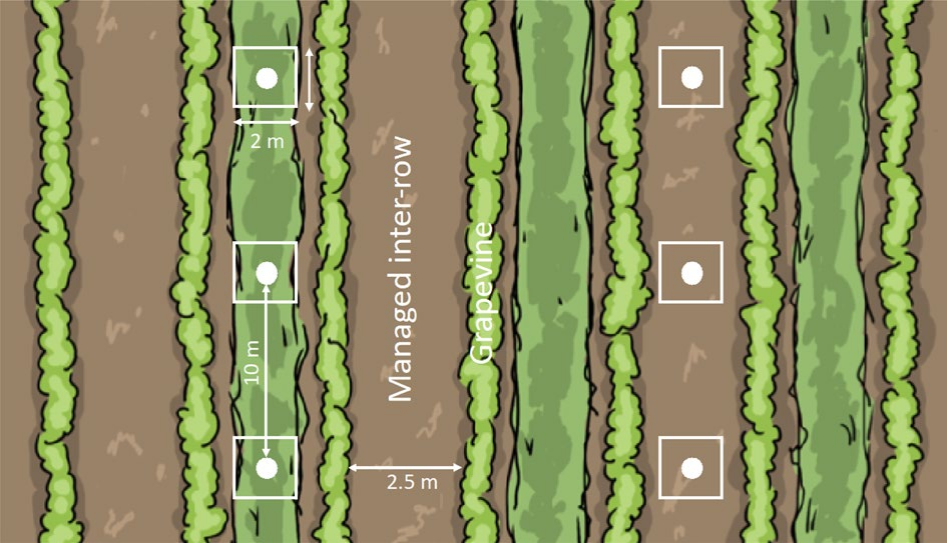
Study design: position of pitfall traps (white circles) (*N* = 6) and vegetation quadrats (white squares) (*N* = 6) in a partially vegetated vineyard (1/2).

A pair of inter-rows separated by two inter-rows was selected in the centre of each vineyard (Fig. 1). The community of plants was studied in three 2 × 2 m quadrats in each selected inter-row from May 15th to June 2nd, resulting in a total of six quadrats per vineyard. The distance between quadrats within rows was 10 m. We identified all vascular plants species and estimated their respective ground cover (%). We then aggregated the cover of each plant species in a group (functional group or family) to obtain the total group cover. We additionally recorded which of the nectariferous species was flowering at the survey date and estimated total flower cover. Plant cover was estimated as the vertical projection of all above-ground organs such that the total vegetation cover may exceed 100%. The cover of flowering nectariferous plants and total vegetation cover were thus estimated separately and do not correspond to the sum of individual species cover.

We assessed the abundance of ground- and canopy-dwelling arthropods using pitfall and sticky traps. One pitfall trap was placed in the middle of each vegetation plot in each vineyard (six pitfall traps per vineyard) (Fig. 1). The traps were 11 cm deep and 8 cm in diameter. They were buried up to the rim and filled with propylene glycol to a quarter of the depth. The epigeic arthropods were collected over two sampling periods (mid-May and mid-June), resulting in a total of 324 traps. Sticky traps consisted of yellow PVC sheets (15 × 20 cm) adhesive on both sides. Five sticky traps were hung to the top wire running alongside the rows of grapevines in each central row between the two inter-rows used for analysis of vegetation (*N* = 135). The lower edge of each trap was 60 cm above the ground and the distance between two traps was 10 m. Sticky traps were set during the second pitfall traps sampling period, i.e. mid-June.

Arthropods were sorted in the laboratory and counted by taxonomic order. We also grouped arthropods according to their diet into: predatory (spiders, harvestman, beetles, lacewing larvae) and phytophagous (hemipterans, beetles, wild bees, lacewing adults) (Appendix 3). As beetles show a great diversity in their trophic regimes, we identified them down to the species level on the basis of taxonomic keys^29,30^ and our personal knowledge, and added two diet groups: omnivorous and detritivorous.

### Data analysis

Generalized linear mixed-effects models (GLMMs) were computed using RStudio (version 1.2.5033) to explore the effects of management type and vegetation characteristics on different response variables (arthropod abundance in pitfall and sticky traps) (package glmmTMB, nbinom2 family for zero-inflated matrix). Arthropod abundance in pitfall and sticky traps were modelled separately. Vineyard (and sampling period only for pitfall traps) was included as random factor.

We modelled separately the effects of inter-row management and vegetation. These two approaches are complementary: models testing the effect of the inter-row management provide information on the effect of the soil management at the vineyard level whereas models testing the effect of vegetation variables provide information on biotic interactions between arthropods and vegetation at a local scale within vineyards.

We modelled the abundance of each arthropod group (beetles, spiders, hemipteran, harvestmen, bees, lacewings) in pitfall and sticky traps according to inter-row management (as a categorical variable) and vegetation variables separately using GLMMs with a negative binomial distribution since these count data were generally overdispersed. We then specifically modelled the abundance of beetles per diet and trap according to inter-row management and vegetation variables. Inter-row management was further tested using the Tukey’s post-hoc test (lsmeans package).

The following variables describing the vegetation structure and community were fitted to the model: (i) vegetation cover, (ii) species richness, (iii) cover of nectariferous flowering plants, (iv) cover of Poaceae species, (v) cover of Fabaceae, (vi) perennial to annual species ratio, and (vii) within vineyard beta diversity calculated using the Bray–Curtis dissimilarity index (R package vegdist) for all pairs of quadrats within vineyards. All vegetation variables (except vii) were recorded at the quadrat level. A stepwise selection procedure using the dredge function was employed to select the most parsimonious model.

We also performed a path analysis (PA) to evaluate the effect of soil management on vegetation parameters and biotic relationships in both ground and canopy components (R package *lavaan*^31^). Path analysis is a specific structural equation modelling (SEM) tool to disentangle relationships between several observed variables^32^. Based on prior knowledge, we developed a conceptual model based upon expected ecological interactions. We assessed the relationships between soil management, the seven vegetation variables (see above), canopy-dwelling arthropods (Hemiptera, wild bees, lacewings, predatory and phytophagous beetles) and ground-dwelling arthropods (spiders, harvestmen, Hemiptera, and phytophagous, detritivorous, omnivorous and predatory beetles). Soil management was coded from 1 for low soil disturbance (fully vegetated vineyards) to 3 for vineyards with periodic mechanical soil management. The full model was simplified by stepwise exclusion of non-significant variables until a minimum adequate model was reached (Appendix 4). The robustness of the final model was determined by checking whether predicted and observed covariance matrices did not differ (χ^2^-squared tests, P > 0.05), whether root mean square error of approximation index was low (RMSEA < 0.1) and whether comparative fit index (CFI > 0.9) and Tucker–Lewis Index (TLI > 0.9) were high. The normal distribution of the residuals of each component model of the full path model was statistically tested. When residuals were not normally distributed, the dependent variables were log-transformed (i.e. abundances of Hemiptera and total predator in the canopy, and abundances of omnivorous and predatory ground-dwelling beetles).

## Results

We collected 14,684 arthropods during the two sampling periods over May and June. Respectively 10,450 and 4234 were collected using pitfall and sticky traps. In pitfall traps, beetles were the most abundant group (4419 individuals), followed by spiders (3099 individuals), Hemiptera (2539 individuals) and harvestmen (393 individuals). Hemiptera were the most abundant group on sticky traps (1815 individuals), followed by beetles (1481 individuals), wild bees (766 individuals) and green lacewings (172 individuals).

### Arthropods abundance according to inter-row management and vegetation

**Figure 2:**
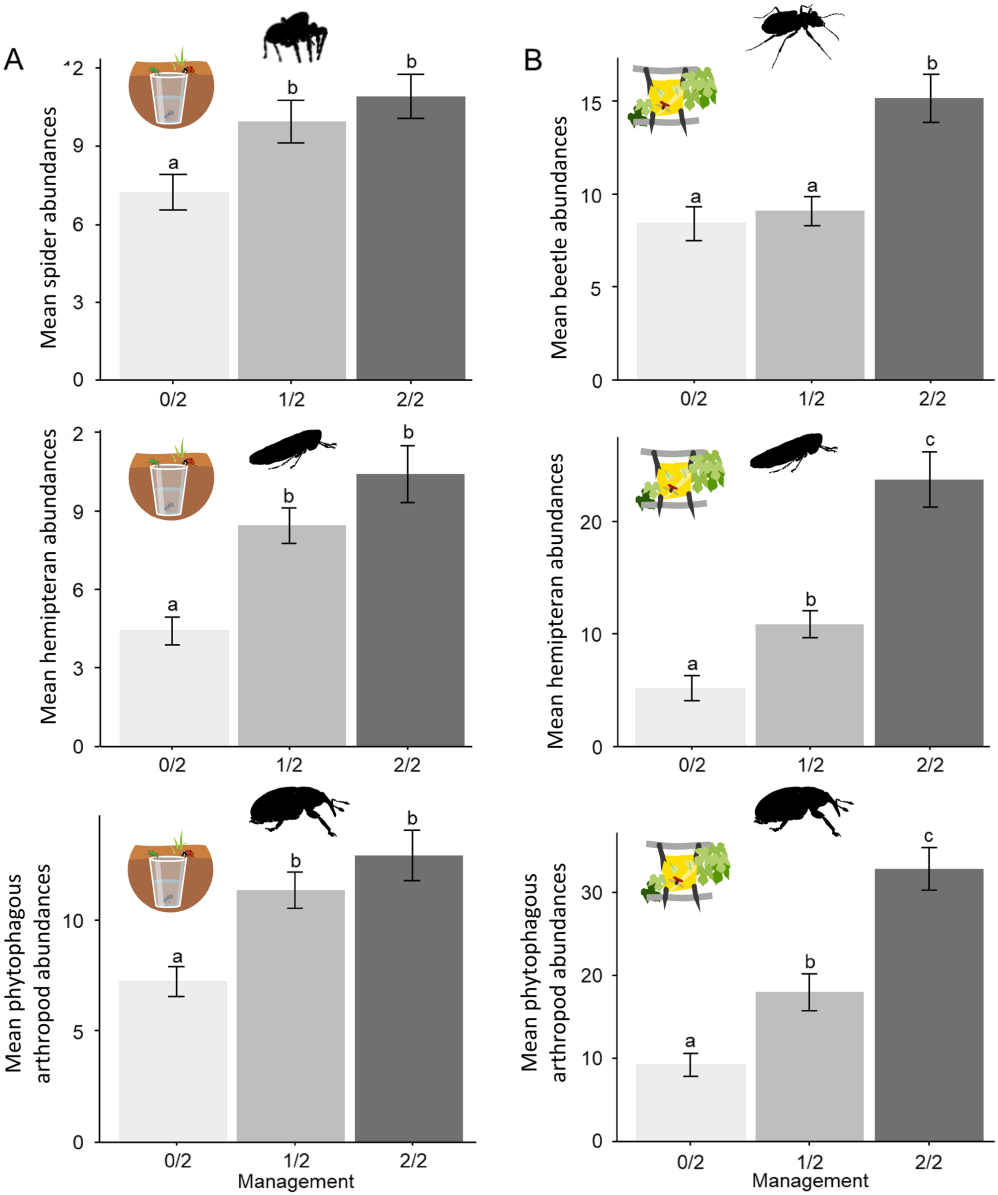
Mean arthropod abundance (± SE) per trap caught using (**A**) pitfall traps and (**B**) sticky traps. The different letters indicate significant differences between inter-row management methods (Post-hoc Tukey tests; P < 0.05).

Overall abundance of arthropods was positively related to plant species richness although the different groups responded differently. In pitfall traps, beetles responded only to the cover of flowering plants, with decreasing abundance as flower cover increased (Table 1). Spiders were significantly more abundant in the 1/2 and 2/2 vineyards than in the 0/2 (Table 1; Fig. 2A). Spider abundance also increased with plant species richness. Similarly, Hemiptera were more abundant in 1/2 and 2/2 vineyards than in 0/2 (Table 1; Fig. 2B). They also responded positively to plant cover (Table 1). The abundance of harvestmen responded positively to plant species richness and negatively to the increase in both flowering plant cover and Bray–Curtis dissimilarity index.

**Table 1:** Effect of inter-row management and vegetation parameters on arthropod and on diet group abundance in pitfall traps.

Dependent variables	Inter-row management			Vegetation cover	Plant richness	Flower cover	Bray–Curtis dissimilarity	Fabaceae cover	Poaceae cover	Perennials/annuals
	0/2–1/2	0/2–2/2	1/2–2/2							
Total arthropods	− 0.26 ± 0.14	− 0.28 ± 0.14	− 0.01 ± 0.13	–	0.02 ± 0.01*	–	– 0.76 ± 0.43.	–	–	–
Beetles	− 0.05 ± 0.2	0.27 ± 0.2	0.32 ± 0.19	–	0.01 ± 0.01.	– 0.03 ± 0.01*	–	–	–	–
Harvestman	0.39 ± 0.58	1.19 ± 0.61	0.81 ± 0.58	–	0.06 ± 0.02**	– 0.13 ± 0.05*	– 3.61 ± 1.74*	–	0.01 ± 0.01.	–
Hemipterans	− 0.76 ± 0.2***	− 1.01 ± 0.2***	− 0.25 ± 0.19	0.01 ± 0.002***	–	–	–	–	–	–
Spiders	− 0.40 ± 0.14*	− 0.55 ± 0.14***	− 0.15 ± 0.13	–	0.03 ± 0.01***	–	– 0.56 ± 0.36	–	0.01 ± 0.002	–
Phytophagous arthropods	− 0.51 ± 0.15**	− 0.72 ± 0.16***	− 0.21 ± 0.15	0.01 ± 0.002**	–	–	–	–	–	–
Predatory arthropods	− 0.29 ± 0.16	− 0.35 ± 0.16.	− 0.07 ± 0.15	–	0.03 ± 0.01***	–	– 0.71 ± 0.43.	–	–	–
Omnivorous beetles	− 0.06 ± 0.76	0.73 ± 0.79	0.79 ± 0.76	–	–	–	–	–	– 0.02 ± 0.01*	− 0.43 ± 0.27
Phytophagous beetles	− 0.07 ± 0.18	− 0.02 ± 0.18	0.05 ± 0.17	–	–	– 0.06 ± 0.02*	0.93 ± 0.60	–	–	− 0.20 ± 0.11.
Predatory beetles	− 0.28 ± 0.29	− 0.15 ± 0.3	0.13 ± 0.28	0.02 ± 0.01**	–	–	–	–0.01 ± 0.01	– 0.01 ± 0.01*	–
Detritivorous beetles	0.23 ± 0.28	0.84 ± 0.28**	0.61 ± 0.27.	–0.005 ± 0.003.	–	–	–	–	–	–

Estimates ± SE of the final models resulting from selection by the "dredge" function for vegetation variables and Tukey posthoc test for inter-row management. 0/2 periodic mechanical soil management to remove vegetation (*N* = 8); 1/2 partially vegetated (every second inter-row is vegetated for a minimum of four years) (*N* = 10); and 2/2 all inter-rows are permanently vegetated (*N* = 9). Values in bold indicate significant effects, codes: 0 ‘***’ 0.001 ‘**’ 0.01 ‘*’ 0.05 ‘.’ 0.1.

**Figure 3:**
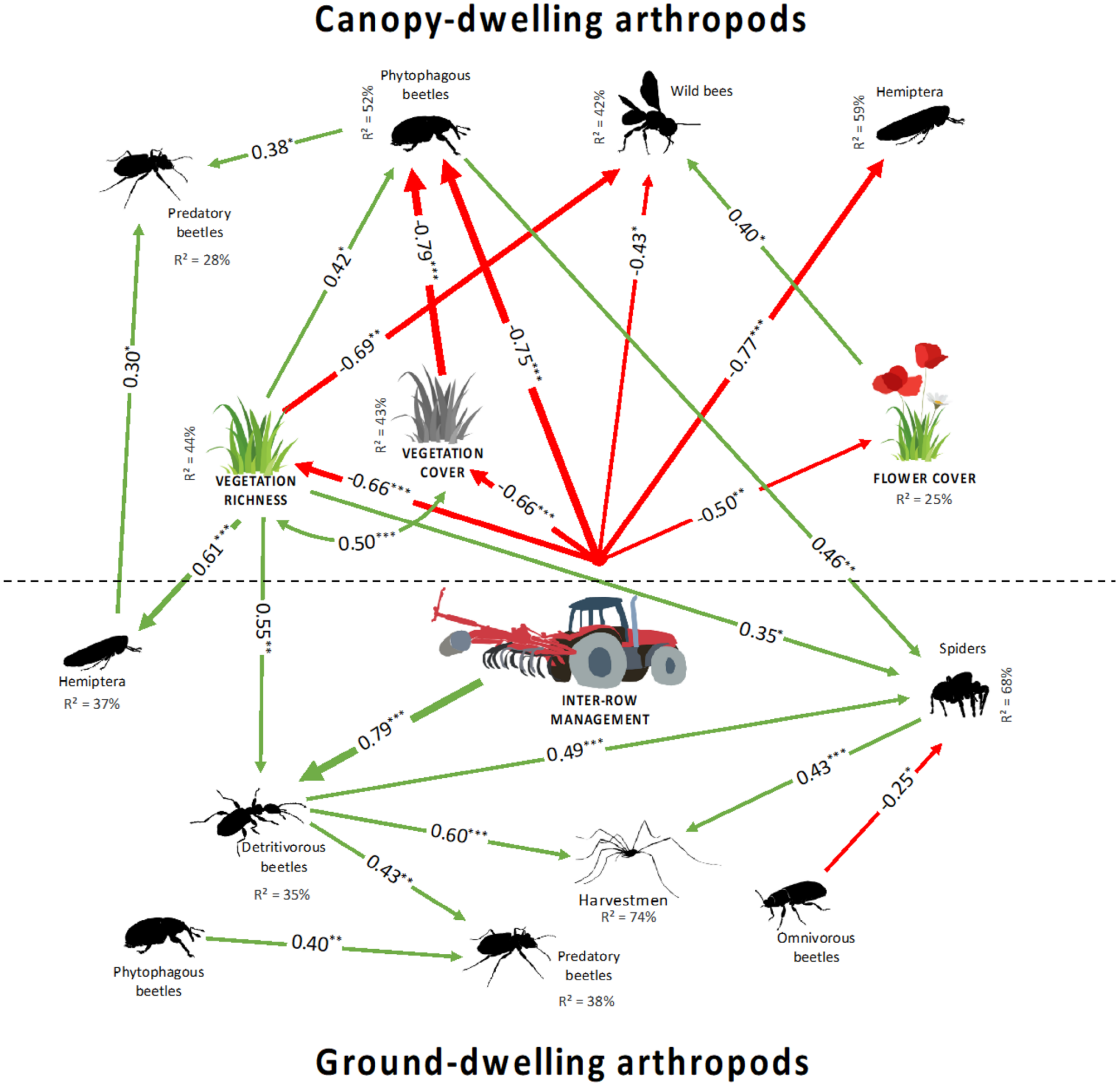
Structural equation model illustrating the strength and direction of the relationships between soil management, inter-row vegetation and arthropods (P = 0.4; RMSEA = 0.04; CFI = 0.99; TLI = 0.98). Green and red arrows denote significant positive and negative effects, respectively. The curved arrow indicates significant co-variation between variables. Arrow widths are proportional to standardised path coefficients that are shown next to the arrows and its significance is denoted as ***P < 0.001; **P < 0.01; *P < 0.05. The strength of the direct paths corresponds to the path coefficient. Percentages indicate the variance explained by the model for each endogenous explanatory variable.

The arthropods sampled by the sticky traps in the canopy were more abundant in fully vegetated vineyards (Table 2). This response was mostly driven by beetles and hemipterans (Table 2; Fig. 3). Both taxa responded also positively to plant species richness (Table 2). Wild bee abundance responded positively to increasing flowering plant cover and perennials/annuals ratio (Table 2).

**Table 2:** Effect of inter-row management and vegetation parameters on arthropod and on diet group abundance on sticky traps.

Dependent variables	Inter-row management			Vegetation cover	Plant richness	Flower cover	Bray–Curtis dissimilarity	Fabaceae cover	Poaceae cover	Perennials/annuals
	0/2–1/2	0/2–2/2	1/2–2/2							
Total arthropods	− 0.41 ± 0.18.	− 1.05 ± 0.18***	− 0.64 ± 0.17***	–	0.06 ± 0.2***	–	–	–	–	0.39 ± 0.2*
Beetles	− 0.08 ± 0.21	− 0.6 ± 0.21*	− 0.52 ± 0.2*	–	0.04 ± 0.01**	–	− 0.99 ± 0.62	–	–	–
Lacewings	0.62 ± 0.48	0.25 ± 0.48	− 0.37 ± 0.47	–	–	–	–	–	–	–
Hemipterans	− 0.84 ± 0.3*	− 1.65 ± 0.3***	− 0.81 ± 0.28*	–	0.1 ± 0.02***	–	–	–	–	–
Wild bees	− 0.54 ± 0.67	− 1.5 ± 0.68.	− 0.96 ± 0.63	–	–	0.32 ± 0.13*	–	–	–	1.3 ± 0.5**
Phytophagous arthropods	− 0.45 ± 0.2.	− 1.18 ± 0.2***	− 0.73 ± 0.19***	–	0.06 ± 0.02***	–	–	–	–	0.44 ± 0.22.
Predatory beetles	− 0.27 ± 0.28	− 0.4 ± 0.29	− 0.13 ± 0.26	–	0.05 ± 0.02**	–	–	–	–	–
Phytophagous beetles	0.01 ± 0.22	− 0.74 ± 0.22**	0.75 ± 0.21**	–	0.04 ± 0.02*	–	–	–	–	–

Estimates (± SE) of the final models resulting from selection by the "dredge" function for vegetation variables and Tukey posthoc tests for inter-row management. 0/2 periodic mechanical soil management to remove vegetation (*N* = 8); 1/2 partially vegetated (every second inter-row is vegetated for a minimum of four years) (*N* = 10); and 2/2 all inter-rows are permanently vegetated (*N* = 9). Values in bold indicate significant effects, codes: 0 ‘***’ 0.001 ‘**’ 0.01 ‘*’ 0.05 ‘.’ 0.1. As the variable "predatory arthropods" was only represented by beetles, it was not included in the table.

### Differential responses of arthropod diet groups

#### Overall arthropod diet groups

In pitfall traps, the abundance of all phytophagous species was higher in the 1/2 and 2/2 vineyards than in the 0/2 (Table 1; Fig. 2A). Phytophagous species responded also positively to the percentage of plant cover. Similarly, on sticky traps, all canopy-dwelling phytophagous species were more abundant in the 2/2 vineyards than in the 0/2 and 1/2 (Table 2; Fig. 2B). In pitfall traps, predators increased in abundance as plant species richness increased (Table 1).

#### Beetle diet groups

The abundance of ground-dwelling phytophagous beetles was negatively correlated with the cover of flowering plants (Table 1). The abundance of ground-dwelling predatory beetles increased with increasing plant cover and decreased with increasing Poaceae cover. Ground-dwelling detritivorous beetles were more abundant in vineyards with no vegetated inter-rows and showed no significant response to vegetation variables. The abundance of ground-dwelling omnivorous beetles only responded to Poaceae cover, with a negative relation (Table 1).

In the grapevine canopy, phytophagous beetles were more abundant in the 2/2 vineyards than in the 0/2 and 1/2 (Table 2; Fig. 2B). The abundance of all phytophagous beetles as well as all predatory beetles increased with plant species richness (Table 2).

### Relationshipss between inter-row management, vegetation and arthropods

The fit of the parameters of the minimal adequate path analysis model (SEM) was very good (P-value: 0.31; RMSEA: 0.05; CFI: 0.98; TLI: 0.97; Fig. 3). We identified a strong link between inter-row management and the vegetation parameters, and between inter-row management and arthropods in the canopy. Plant richness decreased with increasing inter-row management (regression coefficient β = 0.66, P < 0.001) as did the percentage of plant cover (β = 0.66, P < 0.001) and the cover of flowering plants (β = 0.50, P = 0.003) (Fig. 3). Soil management also strongly influenced phytophagous beetles and Hemiptera caught in the grapevine canopy and in to a lesser extent wild bees (Fig. 3). The increase in plant species richness was directly related to an increase in canopy phytophagous beetles but a decrease in wild bees that positively responded to flower cover.

The link between the intensity of soil management and ground-dwelling arthropods was rather indirect through its effects on vegetation, particularly species richness (Fig. 3). Only one relationship was established between the inter-row management and ground-dwelling arthropods, showing an increase in the abundance of detritivorous beetles with an increase in soil management intensity (β = 0.79, P < 0.001). Hemiptera on the ground, detritivorous beetles and spiders were all positively influenced by plant species richness. The three taxa of ground-dwelling predators (spiders, harvestmen and predatory beetles) were positively related to detritivorous beetles, and for spiders additionally to phytophagous beetles in the canopy (Fig. 3).

## Discussion

While the restoration of plant cover in vineyards is increasingly used as a solution to stabilise the soil, prevent erosion and facilitate machinery use, it can also benefit the community of arthropods by offering a wider diversity of micro-habitats. Here we showed that the abundance of arthropods generally increased in vineyards with low intensity of soil management that favoured the development of inter-row vegetation. Plant species richness in particular was found to be positively correlated to the abundance of most groups of arthropods. Moreover, a decrease in inter-row management intensity not only affected each ecosystem component separately (vegetation, soil and canopy fauna) but also interactions between them.

### Differential responses of arthropods to inter-row management and vegetation

Although most of the studied groups showed a positive link to vegetation, three main arthropod responses to inter-row management can be identified. Some arthropods responded linearly to inter-row management intensity, while others showed either a steep decline in periodic mechanically managed vineyards or a steep increase in fully vegetated vineyards.

In our study, the most abundant group was the order of Coleoptera. Interestingly, beetles responded only in the grapevine canopy with higher abundance in fully vegetated vineyards. In the literature, the response of beetles to vegetation and management is also ambiguous. While some studies revealed a positive effect of inter-row vegetation on beetle abundance^18^, others did not find such an effect^33^. As beetles are highly diverse regarding their diet and ecology, we classified them into four groups. The increased abundance of beetles occurring in the canopy of fully vegetated vineyards was driven by the dominance of families that feed on plants such as Mordellidae (393 individuals) and Buprestidae (176 individuals). Canopy-dwelling predatory beetles, that hunt directly on plants and can use plant resources such as pollen and/or nectar as supplementary food, also benefited from increased plant cover. Carabidae and Staphylinidae, the most abundant families in our samples, are known to benefit from the vegetation on the ground^34^. These generalist predators are favoured by the diversification of the agroecosystem and the complexity of resources provided by the vegetation^22^. By their direct and indirect relationships with the vegetation, these species are therefore more likely to benefit from inter-row vegetation^35^.

In contrast, detritivorous beetles showed a preference for vineyards with intensive soil management. Similar results have recently been found in South African vineyards^26^. Species of the Anthicidae family largely dominated this group. Several non-exclusive hypotheses may explain this response. First, such beetles can be locally abundant and may benefit from habitat disturbance, probably by feeding on vegetation litter that is provided by regular soil management in the vineyards. They may be also favoured by a lower level of competition and/or predation regularly observed in disturbed habitats^36,37^. Finally, this increase in Anthicidae abundance might also be the result of a higher probability of capture in pitfall traps. It is known that arthropod abundance data can be influenced by habitat structure, with trapability increasing as habitats become more open^38^. The reduction in habitat complexity in the absence of vegetation may facilitate their movements on the ground, whereas Anthicidae were commonly observed on plants in vineyards with ground cover (pers. obs.).

Hemipterans, largely dominated by leaf hoppers, responded positively to a decrease in soil management both on the ground and in the canopy (Tables 1 and 2). Since leaf hoppers are phytophagous, this result indicated the occurrence of a bottom-up effect with primary consumers favoured by an increase of primary producers^39^ as observed in previous studies^25,33,40^. The increase of phytophagous species in vegetated vineyards may be problematic if it includes pest insects. So far, neither in our vineyards nor in vineyards of other studies^41,42^ such an increase in insect pest infestation in response to increasing inter-row cover has been detected. For example, cover negatively affected pests such as *Panonychus ulmi* and *Scaphoideus titanus* in Bordeaux vineyards (France)^43^. Improved biological control of potential pests by an increase in the number of predators such as spiders favoured by vegetation cover may compensate for potentially positive effects on pest insects^17,44^.

We recorded a clear positive response of spider abundances to a decrease in soil management intensity and an increase in plant species richness (Fig. 3, Table 1). This latter relationship was also observed for harvestmen, whereas harvestmen remained largely unaffected by inter-row management. This is consistent with Vogelweith and Thiéry^43^, who found no effect of inter-row vegetation on *Phalangium opilio*, the most common harvestmen in Bordeaux vineyards. Similar to Anthicidae, harvestmen move on the vegetation, which may reduce their trapability as habitats become more complex. Ground-dwelling predators may use rich inter-row vegetation as a source of prey^45^. This hypothesis is supported by the indirect relationships that we found between spiders and plant richness via an increase in both detritivorous beetles and phytophagous canopy beetle abundances (Fig. 3), groups known to be prey for spiders^46^. Vegetation cover may also offer better microclimate (temperature and/or humidity) facilitating the development and/or reproduction of predators^47^.

Inter-row vegetation provides resources for pollinators, particularly for wild bee species. Several studies have already shown a positive impact of reduced soil management on pollinators^48^. Contrary to Kratschmer et al.^49^, the abundance of wild bee species did not respond to the increase in total plant cover but rather to the increase in flowering plants. A similar pattern has been recently observed between parasitoids and plant cover providing nectar^50^. The relationship confirms the importance of the plant species composition alongside with plant species richness and functional diversity to increase food availability for arthropods. In contrast, lacewings, that have the potential to improve pest regulation, did not show any response to inter-row management or vegetation variables, as already observed in previous publications^51^. For example, Saenz-Romo et al.^52^ found no response of lacewing abundance to treatments [(i) tillage; (ii) spontaneous cover; and (iii) flower cover] in Spanish vineyards. Lacewings change their dietary habits during their life cycle, from generalist predators as larvae to pollinators as adults. Here, we only captured adults, which limits our understanding of the lacewing response to soil management and inter-row vegetation.

### The multi-component effect of inter-row management

The path analysis clearly illustrated the key role of inter-row management intensity in vineyard ecosystems and its major effects on their functioning. Inter-row management and vegetation cover do not only have direct effects on ground-dwelling primary consumers such as detritivorous beetles and phytophagous hemipterans but also on ground-dwelling predators, such as spiders, and on almost all studied canopy-dwelling arthropods. Among the analysed vegetation parameters, plant species richness was by far the most important, with ground cover being less important, but both were only co-variables in the SEM (Fig. 3). Plant richness affected ground- and canopy-dwelling arthropods, from primary consumers to predators, while flower cover only influenced wild bee species, and plant cover only phytophagous beetles. This result suggests that plant species richness, more than vegetation cover, increases the diversity of resources for primary consumers and by makes the habitat more complex, thus offering more ecological niches for arthropods^47^.

This analysis also illustrated the complexity of these agro-ecosystems, with numerous links between different arthropod groups of the grapevine canopy and in the inter-row. Increases in both primary consumers on the ground and in the canopy influenced spider populations in the inter-row. Similarly, predatory beetles in the canopy responded positively to the increase in ground-dwelling hemipterans (Fig. 3). These relationships confirm the movement of individuals from the grapevine canopy to the inter-row and vice versa, an important spill-over effect of inter-row vegetation on the functioning of grapevine agro-ecosystems.

Invertebrates are known to be sensitive to climatic conditions, that change their abundance from year to year^53^. This is particularly true in years of extreme drought and heat that are often associated with lower arthropod abundances. In the short term, such variability may mask the effects of agricultural practices, but major changes such as year-round tillage^1^ or pesticide use^54^, seem be more important in explaining long-term changes in arthropod abundance. Spring 2018 was relatively wet with a peak in temperature and dryness at the end of June when sampling was already completed. Therefore, although we recorded data for one year, the cascading effects of inter-row management we recorded are strong enough to suggest consistency over multiple years despite inter-annual climatic variations.

### Ecosystem restoration in Mediterranean vineyards

Currently, winegrowers are reluctant to maintain or plant vegetation between rows because of competition for water, a particularly important stress factor in water-limited regions such as the Mediterranean^55,56^. However, several studies have demonstrated that inter-row vegetation also provides important ecosystem services^15,21^. In a recent study, we showed that inter-row vegetation improves predation by arthropods^16^. Here, we further highlighted a strong and positive effect of inter-row vegetation on arthropod communities. This positive effect on the abundance of several groups was not limited to ground-dwelling arthropods but was also detected in the grapevine canopy and in trophic relationships between soil surface and canopy. In particular, plant species richness (spiders, harvestmen and canopy beetles) and flower cover (pollinators) had a positive effect on arthropods indicating that highly diverse, flower-rich mixtures are most beneficial to arthropods whereas grass-dominated inter-row vegetation had predominantly negative effects. In a context of growing interest in sustainable and environmentally sound crop management, we showed that the use of plant cover is a key tool to restore arthropod communities in vineyards.

## Supplementary Information

Supplementary Information 1.

Supplementary Information 2.
