# Supplementary material for: Vegetation increases abundances of ground and canopy arthropods in Mediterranean vineyards
Source: Sci Rep. 2022 Mar 7;12:3680. doi: 10.1038/s41598-022-07529-1 (PMC8901849; doi:10.1038/s41598-022-07529-1)
Supplement: Supplementary file 1 — Supplementary Information 1. [file 41598_2022_7529_MOESM1_ESM.docx]

**Appendix 1** Location of analysed wineries in the southern Luberon (south-eastern France). In each winery, one to four vineyards were involved in the study.


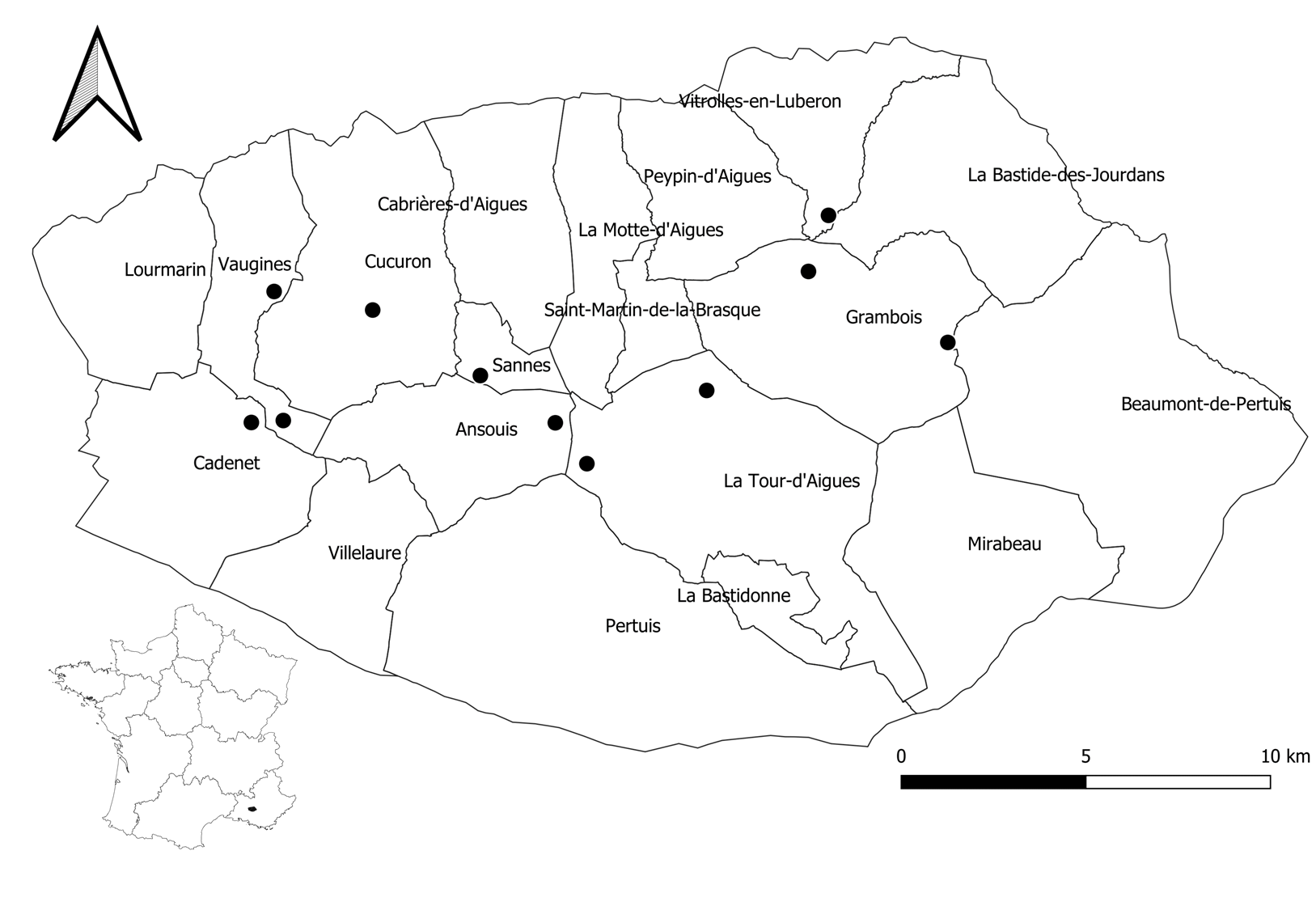


**Appendix 2 Photos of the three types of inter-row management:** (A) periodic mechanical soil management to remove vegetation (shallow tillage) (0/2; *N* = 8), (B) partially vegetated (every second inter-row is vegetated for a minimum of four years) (1/2; *N* = 10) and (C) all inter-rows are permanently vegetated (2/2; *N* = 9) (credits: Chloe Blaise).


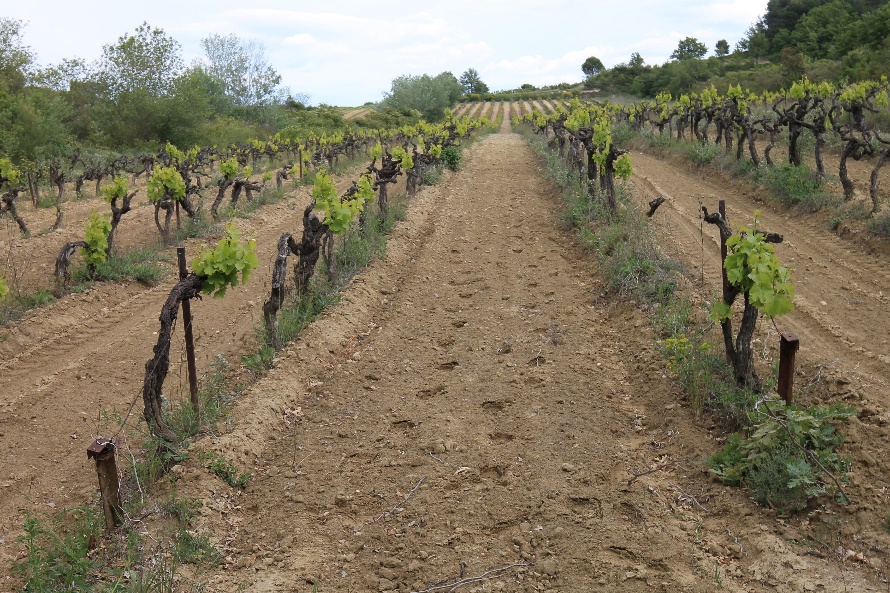

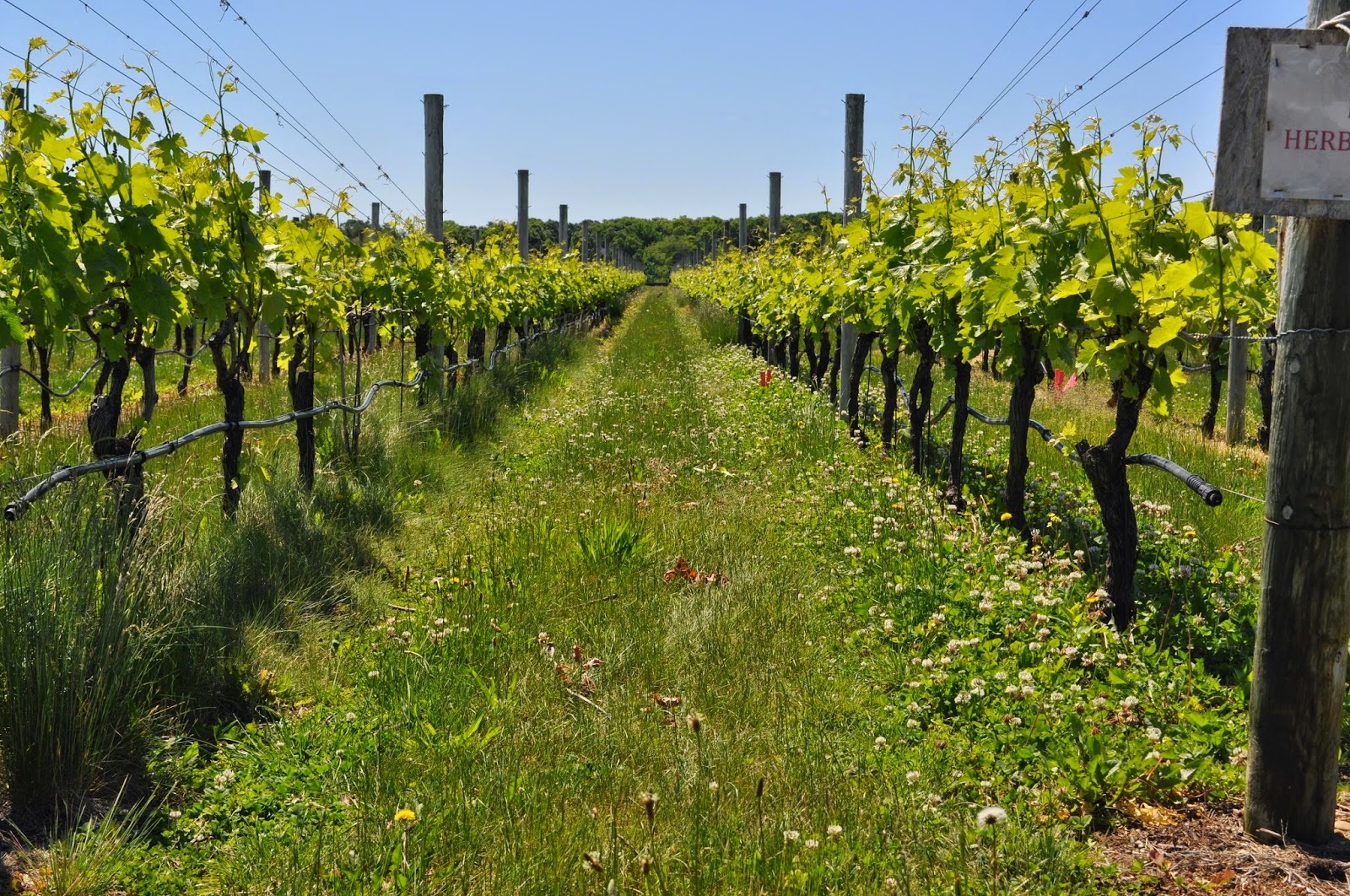

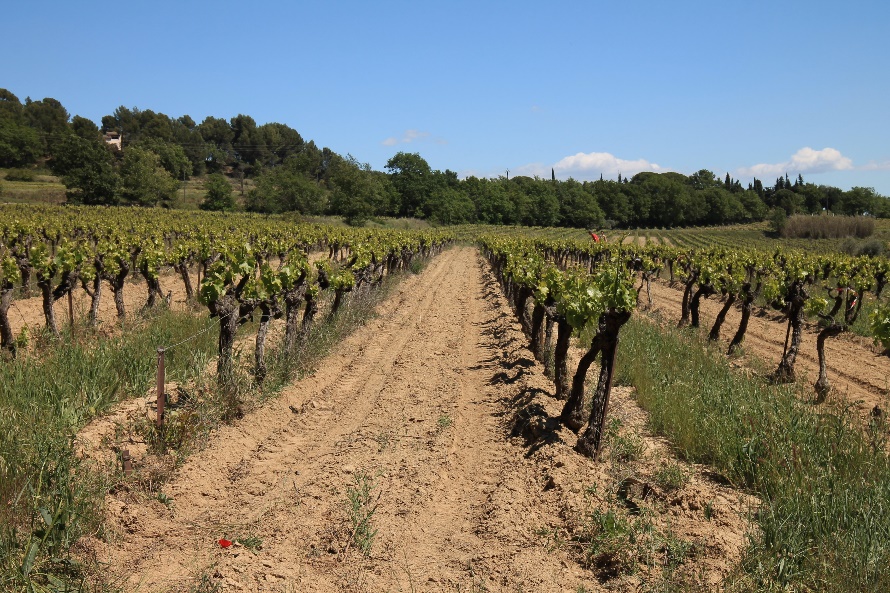


A

B

C

**Appendix 3** Classification of arthropods into ecological groups according to their diet. Beetles were identified to the species level in pitfall traps and to the family level on sticky traps.

**Appendix 4**

Initial conceptual model that was both consistent with our data and made sense biologically. The full model was simplified by stepwise exclusion of non-significant variables until a minimum adequate model was reached. The adequacy of the model was determined by non-significant differences between predicted and observed covariance matrices (chi-squared tests, P > 0.05), low root mean squared error of approximation index (RMSEA < 0.1) and high comparative fit index (CFI > 0.90).

**Model writing:**

dependent_variable_1 ~ independent_variable_1 + independent_variable_2

dependent_variable_2 ~ independent_variable_1 + independent_variable_2

etc.’

**Final model**

Plant richness ~ Inter-row management

Flower cover ~ Inter-row management

Vegetation cover ~ Inter-row management

Vegetation cover ~~ Plant richness

Hemiptera_sticky trap ~ Inter-row management

Bees_sticky trap ~ Flower cover + Plant richness + Inter-row management

Plant Feeder Coleoptera_sticky trap ~ Plant richness + Vegetation cover + Inter-row management

Predatory Coleoptera_sticky trap ~ Hemiptera_Pitfall trap + Plant Feeder Coleoptera_sticky trap

Detritivorous Coleoptera_pitfall trap ~ Plant richness + Inter-row management

Hemiptera_Pitfall trap ~ Plant richness

Araneae_pitfall trap ~ Detritivorous Coleoptera_pitfall trap + Plant richness + Plant Feeder Coleoptera_sticky trap + omnivorous Coleoptera

Opiliones_pitfall trap ~ Detritivorous Coleoptera_pitfall trap + Araneae_pitfall trap

Predator Coleoptera_pitfall trap ~ plant feeder Coleoptera_Pitfall trap + Detritivorous Coleoptera_pitfall trap
